# Energy-Efficient Channel Handoff for Sensor Network-Assisted Cognitive Radio Network

**DOI:** 10.3390/s150818012

**Published:** 2015-07-23

**Authors:** Muhammad Usman, Muhammad Sajjad Khan, Hiep Vu-Van, Koo Insoo

**Affiliations:** Department of Electrical/Electronics and Computer Engineering, University of Ulsan, 93-Daehak-ro, Nam-gu, Ulsan 680-749, Korea; E-Mails: usman@uetpeshawar.edu.pk (M.U.); sajjad.khan@iiu.edu.pk (M.S.K.); vvhiep@gmail.com (H.V.-V.)

**Keywords:** backup channel, energy-efficient channel handoff, imperfect sensing, operating channel, POMDP, spectrum management

## Abstract

The visiting and less-privileged status of the secondary users (SUs) in a cognitive radio network obligates them to release the occupied channel instantly when it is reclaimed by the primary user. The SU has a choice to make: either wait for the channel to become free, thus conserving energy at the expense of delayed transmission and delivery, or find and switch to a vacant channel, thereby avoiding delay in transmission at the expense of increased energy consumption. An energy-efficient decision that considers the tradeoff between energy consumption and continuous transmission needs to be taken as to whether to switch the channels. In this work, we consider a sensor network-assisted cognitive radio network and propose a backup channel, which is sensed by the SU in parallel with the operating channel that is being sensed by the sensor nodes. Imperfect channel sensing and residual energy of the SU are considered in order to develop an energy-efficient handoff strategy using the partially observable Markov decision process (POMDP), which considers beliefs about the operating and backup channels and the remaining energy of the SU in order to take an optimal channel handoff decision on the question “Should we switch the channel?” The objective is to dynamically decide in each time slot whether the SU should switch the channel or not in order to maximize throughput by utilizing energy efficiently. Extensive simulations were performed to show the effectiveness of the proposed channel handoff strategy, which was demonstrated in the form of throughput with respect to various parameters, *i.e.*, detection probability, the channel idle probabilities of the operating and backup channels, and the maximum energy of the SU.

## 1. Introduction

Cognitive radio (CR) technology is a promising solution to prevailing spectrum problems like scarcity and underutilization that vary (between 15% and 85%) according to location and time [1]. In a cognitive radio network (CRN), the secondary users (SUs) opportunistically access the spectrum, which primarily belongs to the primary users (PUs), as long as the frequency bands are temporarily available or the SUs’ transmissions do not generate harmful interference with the PUs. While utilizing the primary network, protection of the PU is the indispensable responsibility of the SU, which can be achieved by performing accurate and timely spectrum sensing.

Typically SUs are capable of spectrum sensing, however, high cost and increased energy consumption make it inappropriate to use them for spectrum sensing alone. A more appropriate approach for improving the sensing performance of a single user involves outsourcing the spectrum sensing to a low-cost dedicated sensor network [2,3,4,5,6], which exploits the location diversity of the sensor nodes and improves sensing accuracy and reliability. It is particularly effective in channels experiencing shadowing, fading, and hidden terminal problems. Like cooperative spectrum sensing [7,8], the sensor nodes perform sensing to determine the status of the PUs locally and send their results to the SU, which combines them using the OR-combination rule. Under the OR-rule, the PU is said to be present if at least one of the sensor nodes reports its presence. Spectrum sensing methods cannot guarantee perfect detection of the presence of the PUs, therefore false alarms and misdetections are unavoidable in real scenarios. A false alarm occurs when a free channel is sensed as busy, whereas a misdetection happens when a busy channel is sensed as free. False alarms result in less utilization of the spectrum (holes) whereas misdetections result in collisions with the PU transmission.

In order to ensure priority channel access for the PU, the less privileged SUs are required to leave the current operating channel (Op-channel) immediately, even in the middle of a transmission, and find a new channel whenever a PU appears on the current channel. This leads to disruptions in transmission and degradation in the quality of service (QoS) for the SU. During spectrum mobility, while protecting the PU, SUs are also required to maintain seamless transmission with minimum disruptions, especially in time-critical (real-time) applications. The spectrum handoff process is an important part of the spectrum mobility: (i) it enables the SUs to maintain transmission without disruption(s) by switching to a vacant channel when the PU re-occupies the current operating channel; and (ii) it ensures efficient utilization of the spectrum holes in the network.

Channel handoff is done using two primary approaches that differ mainly in the time instant of target channel selection [9,10,11], *i.e.*, the proactive (pre-sensing) approach and the reactive (post-sensing) approach. In the proactive approach, a sequence of channels is selected based on the long-term traffic statistics of the channels. This pre-determined sequence is then followed for channel handoff when the PU returns to the current channel. However, in a dynamic environment where the stochastic characteristics of the channels and the PU activity change more often, the old (outdated) information about channels increases transmission collisions between the PU and SUs and also increases “handoff miss” (a handoff is performed but to a busy channel). Therefore, this approach is unlikely to guarantee PU protection and QoS for the SU. In the reactive approach, a vacant channel is selected for handoff through spectrum sensing after the appearance of the PU. The use of the most current information on channels makes this approach less prone to handoff misses and PU collisions and hence more suitable for PU protection and QoS for the SU.

Conventionally, channel handoff occurs if the operating channel is busy; otherwise, the SU operates on the current operating channel. A handoff decision that considers the current status of the operating and other busy channels alone, while ignoring their energy status and future effects, does not provide a long-term reward. The existing literature considers only those situations where SUs search for an alternate or backup channel (Bu-channel) only after the PU reclaims the current operating channel. However, finding a new channel after reappearance of the PU requires extra time, *i.e.*, time required for sensing the channels, choosing an alternate (candidate) channel from among the vacant (sensed as free) channels, and the time required to switch to the candidate channel. A longer delay in the handoff process deteriorates SU transmission and, at times, results in complete disruption. The handoff or switching delay [9] is defined as the duration from the instant that transmission is interrupted until the instant that the unfinished transmission is resumed and the energy consumed in this process is called handoff, or switching, energy. Efficient energy management by the SUs is also an acute challenge, because they operate on a battery with a finite amount of energy. Therefore, the channel handoff decision should also consider energy efficiency (*i.e.*, switch only when it is beneficial) because sensing and subsequent switching consume energy, and hence, affect lifetime and transmission of the secondary network. Energy management of the SU has been studied in [12,13,14], but for a single channel and with no handoff scenarios. Sensing errors are also usually ignored in the channel handoff process, which further deteriorates PU protection and SU transmission.

In this paper, a novel handoff scheme is proposed to address the problems with the existing approaches. The proposed scheme considers imperfect sensing, the energy state of the SU, switching delay, the energy consumed in switching, and channel idle probabilities to answer the question “Should we switch the channel?” The proposed scheme divides the available channels into two groups and performs sensing on both simultaneously, *i.e.*, one group, consisting of the operating channel alone, is sensed using narrowband sensing by the sensor nodes, and the other group, consisting of the remaining channels, is sensed using wideband sensing by the SU. A vacant channel in the latter group is selected as a backup channel. In situations where channel handoff is necessary, re-sensing (time) of the backup channel is not required because its updated information is available. The proposed approach considers imperfect sensing, *i.e.*, inevitable errors in the sensing process, and takes an energy-efficient channel handoff decision using the POMDP framework to maximize the long-term throughput of the SU, considering its energy status along with the idle probabilities (beliefs) of the operating and backup channels. To the best of the authors’ knowledge, no research has considered the parallel existence of operating and backup channels with imperfect sensing and energy status of the SU to make an optimal decision for handoff. The proposed scheme exploits the advantages of both reactive handoff (up-to-date channel status information) and proactive handoff (low handoff delay), thus making it an ideal approach to avoid disruption of SUs’ transmissions and yet guarantee PU protection. The effectiveness of the proposed approach is demonstrated through the simulations presented in Section 5.

The objective of this work is to efficiently utilize energy of the SU and increase its long-term throughput by transmitting either on the operating or backup channel while ensuring PU protection. The main contributions of this work are as follows:

- An energy-efficient spectrum handoff scheme is developed for a sensor network-assisted cognitive radio network, where the SUs are energy-constrained nodes with an energy harvesting capability from the environment.
- The advantages of both reactive and proactive handoff approaches are combined by acquiring up-to-date information on operating and backup channels, thus avoiding extra time to sense a backup channel. The selection of the backup channel is done simultaneously with sensing of the operating channel, thus avoiding delay during channel handoff.
- Imperfect sensing, the energy state of the SU, and idle probability of the channels are considered together and are used to make an optimal handoff decision using the POMDP framework.

The remainder of the paper is outlined as follows: Section 2 gives an extensive literature review. In Section 3, the system model is described in detail, whereas in Section 4, the proposed channel handoff strategy is presented. Results and discussions are presented in Section 5, and the paper concludes with Section 6.

## 2. Related Work

Spectrum handoff is a well-researched topic in cellular communications but it has not been investigated in depth in cognitive radio networks. It is almost unexplored in sensors-assisted cognitive radio networks. Lee *et al.* [15] analytically derived the steady state probability of both handoff and no-handoff cases. The handoff decision is taken considering the current status of the channels alone, ignoring the previous and future (expected) statistics of the channels. The authors in [11] compared proactive and reactive channel handoff strategies in terms of transmission latency by considering different sensing times for a reactive handoff scheme. The primary and secondary user network traffic is described by the preemptive resume priority (PRP) M/G/1 queuing network model. The authors in [9] investigated the effect of spectrum handoffs on channel utilization and data delivery time of the SUs’ connections with various traffic arrival rates and service time distributions. The queuing network model is proposed to characterize channel usage behavior. The authors in [16] proposed a probability approach for the selection of initial and target channels for handoff. Considering the connection-based spectrum handoff, analytical results are provided for the switching-enabled (change or stay) policy of the SUs. The authors in [17] classified the existing handoff approaches and the tradeoff between energy consumption and throughput is described as a function of various parameters, *i.e.*, sensing time, maximum number of handoffs, sensing order, delay, and channel access order. Zhang [18] considered short-term (link maintenance probability and switching delay) and long-term (number of spectrum handoffs and non-completion probability) performance to do spectrum handoff in opportunistic and negotiated scenarios. In the negotiated scenario a spectrum server centrally manages the allocation of channels to both PU and SU. An optimization procedure is developed in [19] to find the channel switching/handoff probability such that the total energy cost comprised of sensing, switching, and transmission energy is minimized. The decision of handoff is based on throughput and delay requirements of the SU.

While analyzing channel handoff, most of the existing literature ignores the energy status of the nodes and/or considers perfect detection of the PU assuming no errors in spectrum sensing. The candidate channel for handoff is selected randomly without any criteria, and the handoff process suffers from conventional delay (*i.e.*, the time for sensing and selecting the best channel and performing handoff after the re-appearance of the PU).

Other researchers [20,21,22,23,24] proposed a backup channel alongside an operating channel for handoff. Han *et al.* [20] proposed an operation mode selection scheme at the cluster head (CH) in a CR sensor network (CRSN). The proposed scheme selects an appropriate mode from among many (operating channel sensing, backup channel sensing, changing the operating channel, changing the backup channel, and data transmission/reception) according to the channel sensing outcome. However, the authors ignore the energy of the nodes and the CH, and randomly choose the backup channel. Furthermore, the proposed method involves the exchange of too many overhead messages between the CH and the CRSN, such as sensing start time, sensing duration, reporting schedule, operation mode decision, and sensing report. The data transmission also involves unnecessary transmission to the CH from the source. A combined framework of routing and channel assignment was proposed in [22] to optimize routing performance and increase network capacity. A backup channel was proposed to avoid end-to-end re-routing. The authors in [21] proposed a spectrum handoff strategy aimed at reducing unnecessary handoffs, considering delay requirements of the application. Using the channel selection algorithm based on a delay violation ratio, the best two channels are chosen, the first (having the minimum delay violation ratio) as a primary channel and the second (the next-to-minimum delay violation ratio) as a backup channel. The backup channel is used in an effort to alleviate errors in channel selection due to an unexpected primary user’s activity. The delay violation ratio is determined as ratio of the number of packets having an estimated delay larger than the delay bound to the total number of packets in the observation window.

The above handoff schemes with a backup channel have not investigated the impact of sensing errors on the primary network. In realistic scenarios, perfect detection of the PU cannot be guaranteed using spectrum sensing techniques. Some authors [23,24] considered imperfect sensing and allocated some channels from the unlicensed bands as backup. A CR switches to the backup channels when the randomly selected licensed operating channels are found busy. Operating on both the licensed and the densely congested unlicensed bands using a single radio interface introduces hardware complexity and more delays in turning from one band to another, as compared to switching from one channel to another in the same frequency band. None of the above studies considered the energy status of the nodes. The backup channel is selected pro-actively (*i.e.*, based on statistical history of the channels), and when the channel handoff decision is taken, then the backup channel is used re-actively (sensed again to get its updated status) by saving only the channel selection time.

Channel handoff is affected by channel selection because the selection of a common channel for handoff by many SUs results in collisions between their transmissions. The channel selection algorithm also needs to be fast enough to select a proper channel as a backup before the handoff process initiates. Hence, various channel selection techniques have been investigated. Ma *et al.* [10] proposed a handoff scheme based on POMDP to select the optimal target channel for spectrum handoff according to partially observable channel state information. A recommended channel sequence (RCSS) is generated in [25] considering non-identical signal-to-noise ratios (SNRs) of the PU signal on different channels. Mishra *et al.* [26] proposed three channel selection techniques based on different constraints, *i.e.*, minimum number of channel switches, maximize overall throughput, and a combined value between minimal channel switching and maximal throughput. In [27], a distributed channel selection scheme is proposed to eliminate collisions among SUs in a multiuser spectrum handoff scenario. The authors proposed a framework in which the SUs coordinate with each other without a common control channel. Moreover, a probability-based prediction method was proposed for channel switching. In this work, the channel selection procedure in [27] is adopted and primary focus is placed on an energy-efficient channel handoff. A real-time control/scheduling algorithm [28] may also be adopted for channel selection.

## 3. System Model

### 3.1. Assumptions and System Preliminaries

**Figure 1:**
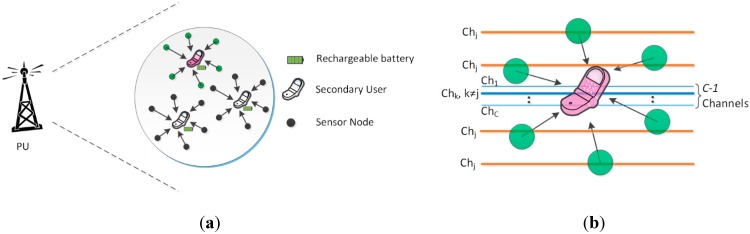
(**a**) Sensors-assisted cognitive radio network operating in the coverage area of the primary network. PU represents the PU-transmitter; (**b**) Sensor nodes sensing the operating channel, and the SU sensing the C−1 channels.

This study considers a sensors-assisted cognitive radio network (as shown in Figure 1a) acting as a secondary network in the domain of a primary network. Each SU is assisted by N−1 sensor nodes for spectrum sensing. The primary network consists of *C* channels that can be shared between the PU and the SUs. Sensor nodes perform narrowband spectrum sensing of the operating channel, which is specified by the SU, whereas the SUs perform wideband spectrum sensing [29,30] of the remaining C−1 channels. Wideband sensing is used to sense multiple channels simultaneously and to select one of the free channels as a backup channel. The operating and backup channels are shown in Figure 1b.

**Figure 2:**
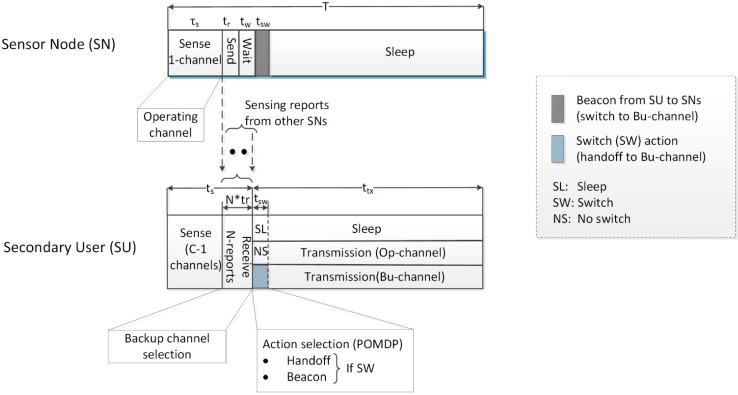
Slot structure of the sensor nodes and secondary user.

The considered system consists of three entities, *i.e.*, the primary network, the sensor network, and the cognitive radio network. PU activity is assumed to be time-slotted on each channel and is modeled by the discrete-time Markov chain. The state space of the Markov chain consists of two states: busy (B) and free (F). The busy state shows the presence of the PU signal, *i.e.*, the channel is busy, and the free state represents the absence of the PU signal, *i.e.*, the channel is free. It is assumed that the state transition probabilities $P_{i}^{FF}$ (from state F to itself) and $P_{i}^{BF}$ (from state B to F), where i={O(operating channel), B (backup channel)}, are known to the SU through estimation based on long-term spectrum usage measurements [31]. For simplicity, it is assumed that PU characteristics are the same on all channels sensed by the SU [15,17,19]. The sensor nodes perform in-band spectrum sensing of the operating channel, send their reports to the SU, wait for the channel handoff decision (*i.e.*, beacon message from the SU), and then sleep until the next sensing slot in order to conserve energy. The wait period is denoted by *t_w_* and is approximately equal to the total reporting period of the sensor nodes clustered with a SU. The sensor nodes switch to a new operating channel (the old backup channel) upon receiving a control (beacon) message from the SU after the wait period, when a channel handoff decision is taken by the SU, as shown in Figure 2. This means the operating channel does not remain constant; it may change during operation of the sensor nodes.

The SU is a battery-operated energy-harvested node that performs out-band sensing of multiple channels and selects one of them as a backup channel. It is assumed that the SU always has data to transmit. A slotted operation of the SU is assumed where each slot mainly consists of sensing and transmission periods. The sensing period of the SU consists of wideband spectrum sensing and receiving reports from the sensor nodes. The SU, while receiving and fusing reports it receives from the sensors, concurrently runs the selection process for the backup channel on the result of wideband sensing. Thus, by the end of the reporting period, the SU knows the status of both the operating and backup channels and is able to take an appropriate action. The amount of time consumed in channel and action selection is assumed to be negligible because of the common availability of reasonably high power computational nodes. After knowing the status of the operating and backup channels, the SU makes an optimal decision answering the question “Should we switch the channel?” using the POMDP framework, considering its energy state and beliefs about the operating and backup channels. Therefore, it either remains in sleep mode, continues transmission on the operating channel, or switches to the backup channel and continues transmission. The detailed slot structure is shown in Figure 2. When the SU decides to handoff the current channel, it broadcasts the decision to the sensor nodes through a beacon message and performs handoff by tuning its radio to the backup channel, consuming energy $e_{sw}$. The SU is able to transmit data for the duration of $t_{tx}-t_{sw}$ if the backup channel is free.

The signaling overhead between the sensor nodes and the SUs is very minimal because of the clustering approach. Due to the formation of clusters and then sub-clusters (subsets) [5], the number of sensor nodes that are actually involved in signaling with the SU is quite small with negligible effects. Moreover, since the sensor nodes send their reports as single-bit hard decisions to the SUs, the signaling overhead from sensor nodes to the SUs is very low. Similarly, the signaling overhead from the SUs to the sensor nodes is also very low because the beacon is a very short message and is broadcasted by an SU to the sensors nodes only if a switch decision is being taken.

### 3.2. Spectrum Sensing

In each slot, the SU performs wideband spectrum sensing during the sensing slot to obtain the status of the *C − 1* channels, and the sensor nodes perform narrowband sensing on the operating channel (denoted by the *j*-th index). The received signal at the channels, using the energy detection technique, is a binary hypothesis testing problem and is given as

(1) $$\begin{array}{l} H_{0,k}^{l}:x_{k,l}(n)=u(n),\\ H_{1,k}^{l}:x_{k,l}(n)=h_{k,l}(n)s(n)+u(n),\;n=1,2,...,S\; \end{array}$$

where l shows index (1,2,…,N) of the sensor nodes and SU in which index *N* is used for the SU, k represents index (1,2,…,C) of channels in which index *j* is used for the operating channel, $H_{0,k}^{l}$ and $H_{1,k}^{l}$ corresponds to the hypotheses that the PU signal is absent and present on k-th channel at *l*-th sensor/SU, respectively, s(n) represents the primary signal received at the channel, h(n) is gain of the channel, u(n) is the additive white Gaussian noise (AWGN) with zero-mean and ${\text{σ}}_{u}^{2}$-variance, and S is the number of samples that is equal to the time bandwidth product. We assume that s(n) and u(n) are completely independent. Without loss of generality, the variance of noise is assumed to be the same. The energy observed on the *k*-th channel is given by

(2) $$Y_{k}^{l}=\sum_{i=1}^{S}{\left|x_{k,l}(n)\right|}^{2}$$

For the SU, k=1,2,…,C with k≠j and l=N, whereas for the sensor nodes, k=j and l=1,2,…,N−1. The local decision about the PU state on each channel is given as

(3) $$Y_{k}^{l}\overset{{\hat{H}}_{1,k}^{l}}{\underset{{\hat{H}}_{1,k}^{l}}{\frac{>}{<}}}\lambda$$

where λ is the energy threshold for the local decision, ${\hat{H}}_{0,k}^{l}=0$ and ${\hat{H}}_{1,k}^{l}=1$ are the sensed-as-free and the sensed-as-busy states, respectively, of the *k*-th channel and *l*-th sensor/SU. According to the central limit theorem (CLT), for a larger value of S, $Y_{k}^{l}$ follows a Gaussian distribution, *i.e.*, $Y_{k}^{l}∼N\left(\text{Ε}\left(Y_{k}^{l}\right),V\left(Y_{k}^{l}\right)\right)$, under both hypotheses [32]. The mean ($\text{Ε}\left(Y_{k}^{l}\right)$) and variance ($V\left(Y_{k}^{l}\right)$) of $Y_{k}^{l}$ are given, respectively, as

(4) $$E(Y_{k}^{l})=\left\{ \begin{array}{l} H_{o,k}^{l}:S\sigma_{u}^{2}\;\\ H_{1,k}^{l}:N(S(\gamma_{l}+1)\sigma_{u}^{2} \end{array}\right.$$

(5) $$V(Y_{k}^{l})=\left\{ \begin{array}{l} H_{o,k}^{l}:2S\sigma_{u}^{4}\;\\ H_{1,k}^{l}:2S(2\gamma_{l}+1)\sigma_{u}^{4} \end{array}\right.$$

where ${\text{γ}}_{l}$ is the SNR of the primary link at the *l*-th sensor/SU. As discussed earlier, in real scenarios there are always sensing errors that cause false alarms and misdetections. Considering the imperfect sensing, the probability of detection is defined as the probability that an SU correctly detects the presence of the PU signal. On the other hand, the false alarm probability is defined as the probability that an SU detects the PU incorrectly, *i.e.*, the PU is absent, in fact, but the SU detects otherwise. For better utilization of the primary channel and more protection of the PU, the SU is required to have a lower value of the false alarm probability and a higher value of the detection probability, respectively. The detection probability and false alarm probability, respectively, at the *l*-th node and *k*-th channel are expressed mathematically as

(6a) $$P_{d,k}^{l}=P(Y_{k}^{l}\geq \lambda |H_{1,k}^{l})=Q\left(\frac{\lambda -S(\gamma_{l}+1)\sigma_{u}^{2}}{\sigma_{u}^{2}\sqrt{2S(2\gamma_{l}+1)}}\right)$$

(6b) $$P_{f,k}^{l}=P(Y_{k}^{l}\geq \lambda |H_{0,k}^{l})=Q\left(\frac{\lambda -S\sigma_{u}^{2}}{\sigma_{u}^{2}\sqrt{2S}}\right)$$

where Q(.) is the complementary cumulative distribution of the standard Gaussian. The value of the threshold λ affects the probability of detection and the probability of false alarm. A higher threshold results in a smaller probability of false alarm but a larger probability of misdetection. Note that, because of the earlier assumption that PU characteristics on multiple channels at the SU are same, we can write the probability of detection and probability of false alarm, respectively, of the channels at the SU in a time slot as

(7a) $$P_{d}^{B}=P_{d,1}^{l}=P_{d,2}^{l}=...=P_{d,C-1}^{l}$$

(7b) $$P_{f}^{B}=P_{f,1}^{l}=P_{f,2}^{l}=...=P_{f,C-1}^{l}$$

Since we are using the OR fusion rule at the SU for the received reports from the sensor nodes, the global detection probability and the global false alarm probability, respectively, of the operating channel are

(8a) $$Q_{d,j}=1-\prod_{l=1}^{N-1}(1-P_{d,j}^{l})$$

(8b) $$Q_{f,j}=1-\prod_{l=1}^{N-1}(1-P_{f,j}^{l})$$

### 3.3. Throughput and Energy Harvesting Model

#### 3.3.1. Throughput

The SU can transmit only if either the operating channel or the backup channel is free. If the operating channel is busy, an optimal action to switch or not to switch is taken by the POMDP. We consider the case of imperfect sensing, where transmission between the SU transmitter and receiver will occur only if the PU is actually absent. This is ensured through the channel state information (CSI) by waiting for an acknowledgement (ACK) message from the SU receiver. If the ACK message is not received within a due time, timeout occurs and the transmission is assumed to have collided with the PU, thus resulting in no throughput. Also, throughput cannot be achieved if the SU stays idle or if both the operating and backup channels are sensed as busy. On the other hand, if the ACK message is received within the pre-defined time, the transmission is considered successful and throughput is achieved. The standard throughput of the secondary link is given as $C=log_{2}\left(1+\text{Γ}\right)$ bits/s/Hz, where Γ is the SNR of the secondary link. It is assumed that the channel gain and the SNR between the SU transmitter and receiver are known to the transmitter through feedback from the receiver. The average throughput on the operating and backup channels, respectively, are given as

(9a) $$R_{Op}=\frac{\left(T-t_{s}\right)}{T}C(1-Q_{f,j})P(H_{0,j})$$

(9b) $$R_{Bu}=\frac{\left(T-t_{s}-t_{sw}\right)}{T}C(1-P_{f}^{B})P(H_{0,B})$$

where T is the slot duration, $t_{s}$ is the sensing duration, $t_{sw}$ is the switching time, and $P(H_{0,j})$ and $P(H_{0,B})$ are the steady state probabilities of the operating and backup channels, respectively, in the *F*-state [31]. Due to imperfect sensing and staying in sleep mode in some slots because of energy constraints and/or the channels' states, the achieved throughput will always be less than the ideal throughput (which is achieved by considering perfect sensing, infinite energy of the SU, and no PU transmission).

#### 3.3.2. Energy Harvesting Model

Efficient energy management is critical for sustaining the lifetime of the network. We assume that the SU is powered by a finite capacity battery that can be recharged by the energy harvester, which harvests energy from ambient sources. Energy harvesting can be, broadly, divided into two categories: radio-frequency (RF) energy harvesting that harvests energy from ambient electromagnetic energy [33,34,35,36], and non-RF energy harvesting, which can include photo voltaic cells for harvesting solar energy or electrostatic or electromotive devices for harvesting energy from sources of mechanical vibrations or acoustic waves [37,38,39]. Chen *et al.* [35] proposed an adaptive energy beamforming technique to jointly maximize wireless energy harvesting and information transfer. Wan *et al.* [40] presented a combined study of energy management and energy harvesting for the sensor network, and illustrated it with a case study. Harvesting energy from solar, wind, and thermal sources can be done both on the macro and micro scales. On the macro scale, it generally requires large infrastructure and is used for large-scale applications. However, on the micro scale, it only requires an array of micro-scale devices fabricated on silicon substrates, which have been successfully used and applied in sensor networks, and portable and hand-held devices. For example, there are devices available that scavenge solar energy for mobile devices [38]. There are plenty of technologies and devices that are currently available on the market, and more are being developed, which can be used in the context of our application, *i.e.*, recharging the SU’s battery [41,42,43]. The harvested energy from these sources can be used to recharge the battery of the SU directly or the harvested energy is stored in a separate battery or in very high capacity condensers, *i.e.*, capacitors that can power the SU on demand.

The SU is able to perform other operations (*i.e.*, sensing, receiving, transmitting, and processing, *etc.*) in parallel with energy harvesting. The SU can harvest $e_{h}(t)\in {\text{E}}_{h}=\{e_{1}^{h},e_{2}^{h},\cdots e_{m}^{h}\}$ packets of energy, but the energy of the SU cannot exceed the maximum capacity of the battery, $e_{max}$ , *i.e.*, $e\left(t+1\right)=e\left(t\right)+e_{h}\left(t\right)\leq e_{max}$. It is assumed that the harvested energy packets follow the Poisson process with mean $e_{h\mu }$ [36,44]. The probability distribution of the harvested energy is given as

(10) $$\mathrm{Pr}(e_{h}(t)=i)=e^{-e_{h\mu }}\frac{{\left(e_{h\mu }\right)}^{i}}{i!}\;i=1,2,3,...,m$$

Because sensing, switching, and transmission processes consume energy, the SU checks the existing energy, at the beginning of each time slot, to determine whether it is enough to carry out the corresponding operation or not. The available energy for the next slot is the energy remaining after sensing, transmission, handoff, and harvesting, and is given as

(11) $$e(t+1)=\min(e(t)+e_{h}(t)-(e_{tx}+e_{s}+e_{sw}),e_{\max})$$

where $e_{tx},\;e_{s},\;e_{sw}$ are the energy required for transmission, sensing, and switching, respectively. Note that $e_{tx}+e_{s}\leq e\left(t\right)$ (when the SU decides to operate on the operating channel) and $e_{tx}+e_{s}+e_{sw}\leq e\left(t\right)$ (when the SU decides to operate on the backup channel).

## 4. Proposed Handoff Strategy

### 4.1. Operation Modes/Actions

The SU operates in different modes as a result of the actions made by POMDP, which depend on the status of the operating and backup channels and the energy status of the SU. Harvesting energy is independent of the SU modes, whereas the consumption of energy is strictly dependent upon operating modes.

*Sleep (SL)*: In the sleep mode, the SU transmission circuitry is turned off in order to conserve energy, whereas the harvester is able to harvest energy. This mode occurs as a result of insufficient energy to carry out transmission and/or switching or the busy status of both the operating and backup channels. In this mode, energy is not consumed nor is throughput achieved.

*No Switch (NS)*: In this mode, the SU stays on the operating channel and transmits data if the operating channel is free. This action infers that the remaining energy of the SU is not enough to carry out channel handoff and transmission, or the operating channel is sensed as free, or both the operating and backup channels are sensed as busy. Throughput will be achieved if ACK is received from the SU receiver on the operating channel. In this mode, energy is consumed in sensing and/or transmission.

*Switch (SW)*: In this mode, the SU switches from the operating channel to the backup channel by consuming the switching energy, and generates the beacon message for the sensor node to switch in the next slot. This action implies that the SU has enough energy to perform switching and transmission, and the operating channel is sensed as busy whereas the backup channel is sensed as free. In this mode, energy is consumed in sensing, handoff, and subsequent transmission. Throughput will be achieved if ACK is received from the SU receiver after transmission on the backup channel.

**Figure 3:**
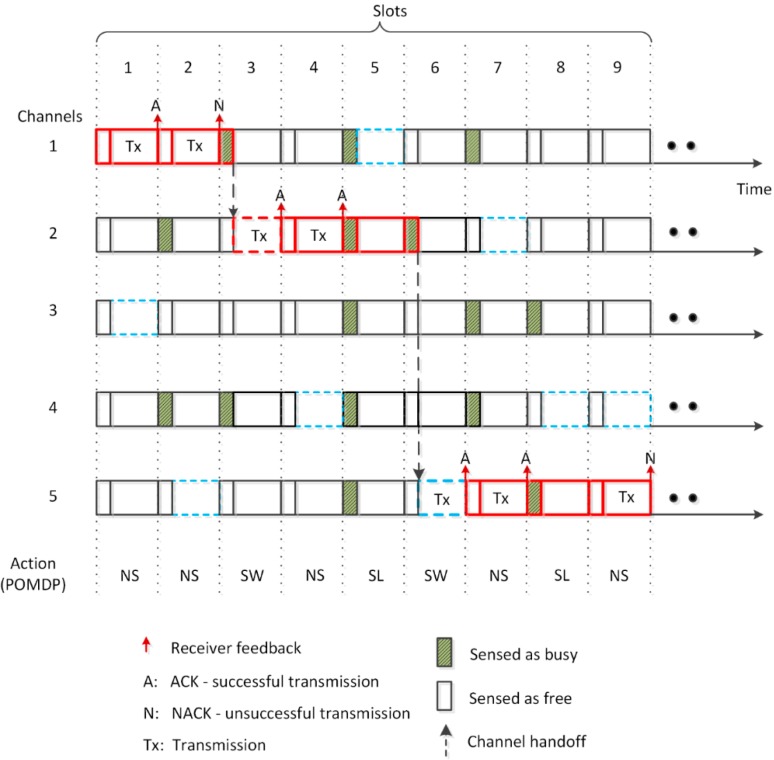
Operation of the proposed system.

### 4.2. System Operation

In each time-slot, the SU performs sensing of the *C-1* channels and selects one amongst them as the backup channel. At the same time, the sensor nodes perform sensing of the operating channel. The POMDP framework, based on the beliefs about the operating and backup channels and the energy of the SU, takes an appropriate action in each time slot. The SU continues to transmit on the operating channel, if it is available, because channel switching incurs delay and energy costs upon the SU and the sensor nodes. The operation of the SU is illustrated in Figure 3. In the first time slot, the SU selects channel 3 as the backup channel, while channel 1 is the already selected operating channel. Since the action decided by POMDP is NS, the SU continues transmitting on the operating channel which is acknowledged (ACK) by the receiver. This sequence is repeated for the second time slot but the backup channel changes to channel 5. The SU continues to transmit on channel 1, however, due to the ACK timeout, no throughput is achieved. In the third time slot, channel 2 is chosen as the backup channel which is sensed as free, whereas the operating channel is sensed as busy. The POMDP selects action SW which forces the SU to perform handoff and broadcast a beacon message to the sensor nodes to switch their radios to channel 2, the operating channel for the next time slot. Channel 1 becomes a candidate for the backup channel in future time slots. In the fourth time slot, the SU continues to operate on channel 2 as the selected action is NS. In the fifth time slot, POMDP selects SL action as all channels are sensed busy. The SU, therefore, stays on channel 2 but does not transmit. In the sixth slot, the SU leaves channel 2 and switches to channel 5 (new backup channel) where it transmits until the seventh time slot as decided by the POMDP. In the eight time slot, the SU neither switches to the free backup channel (channel 4) nor transmits, as POMDP chose action SL due to insufficient energy of the SU for either switching or transmitting. The operating channel is shown in a thick red border, whereas the backup channel is shown in a dashed blue border. Notice that both the operating and backup channels switch roles during operation, *i.e.*, each time the SU switches to a backup channel, it becomes the new operating channel, whereas the previous operating channel becomes a candidate backup channel for future time slots.

### 4.3. Should We Switch the Channel? A POMDP-Based Optimal Action Selection

The POMDP framework is used to determine an optimal mode selection (action) policy based on the partially available information and observations from the system. Generally, POMDP uses observations from the environment, action, current state, and belief state to estimate a future state. Based on the belief state, the optimal action is selected, which results in observation of the environment.

*Action space:* At the start of each time slot, the SU determines an action a(t) based on the residual energy and belief about the operating and backup channel states. An action results in one of the operation modes of the SU: sleep (SL), no switch (NS), and switch (SW). Therefore, the action and operation modes are used interchangeably. The action space A consists of all possible actions, which are given as a(t)∈A={SL,NS,SW}.

*State space:* The state of the system, denoted by s(t), in the current time slot is defined as

(12) $$s(t)=\{e(t),p_{O}(t),p_{B}(t)\}$$

At the start of each time slot, the SU has beliefs, $p_{O}\left(t\right)$ and $p_{B}\left(t\right)$, about the operating channel and backup channel, respectively. Belief about a channel is referred to as the probability that the PU is absent on that channel in the time slot. We assume that the initial belief is approximately equal to the idle probability of the channel. Belief state has sufficient statistical data about all past actions and observations necessary for decision-making under environmental uncertainties. Depending on the action and corresponding outcome, beliefs are updated accordingly to incorporate additional information about the environment into the history.

*Reward:* In order to ensure optimal action by the POMDP, each action is accompanied by a reward or a penalty, R(s(t),a(t)). Reward is the throughput of an SU link when successful transmission occurs between the SU transmitter and receiver, whereas the penalty is zero throughput, which occurs in sleep mode or transmission without ACK, *i.e.*, negative ACK or NACK. Immediate reward is obtained when action a(t) is taken, which makes a transition from the current state s(t) to the next state s(t+1) with observation ϕ. The choice of action is stimulated by enhancing the value function $V(e\left(t\right),p_{O}\left(t\right),p_{B}\left(t\right)$) which is defined as the maximum total discounted throughput from the current slot when the remaining energy is e(t) and the belief about the free state of the operating and backup channels is $p_{O}\left(t\right)$ and $p_{B}\left(t\right)$, respectively. The expression of the value function is given below:

(13) $$\begin{array}{l} V(e(t),p_{O}(t),p_{B}(t))=\\ \underset{a(t),a(t+1)...\in A}{\max}Ε\left\{\sum_{t=k}^{\infty }\left(\delta^{t-k}R\left(e(t),p_{O}(t),p_{B}(t),a_{i}(t)|e(k),p_{O}(k),p_{B}(k)\right)\right)\right\} \end{array}$$

where 0 ≤ δ < 1 is the discount factor which is used to value the current reward more than future rewards.

*Observation space:* In a partially observable environment, a set of observations is used to estimate the state of the environment. Considering the beliefs and energy state, the SU takes the optimal action using the POMDP framework and further updates the beliefs, energy, and other parameters for the next time slot. Details of all possible actions and observations of the channel states are presented below.

#### 4.3.1. Sleep Mode (a(t)=SL):

If the sleep mode is decided by the SU, it would not be able to gain any throughput, *i.e.*, $R(e(t),p_{O}(t),p_{B}(t),SL)=0$. In this mode, the SU updates its belief about the operating and backup channels, respectively, being free in the next slot as follows:

(14a) $$p_{O}(t+1)=p_{O}(t)P_{O}^{FF}+(1-p_{O}(t))P_{O}^{BF}$$

(14b) $$p_{B}(t+1)=p_{B}(t)P_{B}^{FF}+(1-p_{B}(t))P_{B}^{BF}$$

The existing energy is updated to $e\left(t+1\right)=\text{min}\left(e\left(t\right)+e_{h}\left(t\right),\;e_{max}\right)$. The probability of the energy update, given the observation (transition probability) is

(14c) $$\mathrm{Pr}(e(t)\to e(t+1);SL)=\mathrm{Pr}[E_{h}=e_{h}(t)]$$

#### 4.3.2. No Switch Mode (a(t)=NS):

If the SU decides on the no-switch mode, it achieves throughput depending on observation of the channels. Considering the imperfect sensing and status of the channels, the following three observations are possible in this mode.

*Observation 1* $\left({\text{ϕ}}_{1}\right)$*:* The operating channel is sensed as free. The SU transmits data and receives an ACK message from its corresponding receiver, confirming the successful transmission of data due to the actual absence of the PU. The state of the backup channel is “don’t care”, which means the SU will transmit on the operating channel if it is free, irrespective of the backup channel state. The throughput achieved in this case is given as

(15a) $$R(e(t),p_{O}(t),p_{B}(t),NS,{\text{ϕ}}_{1})=\frac{T-\tau_{s}}{T}C$$

The updated belief of the operating and backup channels, respectively, are given as

(15b) $$p_{O}(t+1)=P_{O}^{FF}$$

(15c) $$p_{B}(t+1)=\left\{ \begin{array}{l} p_{B}(t)P_{B}^{FF}\text{, }if\;{\hat{H}}_{0,B}^{N}\;\\ (1-p_{B}(t))P_{B}^{BF}\text{, }if\;{\hat{H}}_{1,B}^{N} \end{array}\right.$$

where ${\hat{H}}_{0,B}^{N}$ and ${\hat{H}}_{1,B}^{N}$ represent the free and busy states, respectively, of the backup channel. Energy of the SU is updated to $e\left(t+1\right)=min\left(e\left(t\right)+e_{h}\left(t\right)-e_{s}-e_{tx},\;e_{max}\right)$. The observation and transition probabilities, respectively, are given as

(15d) $$\mathrm{Pr}({\text{ϕ}}_{1})=p_{O}(t)(1-Q_{f,j})$$

(15e) $$\mathrm{Pr}(e(t)\to e(t+1)|{\text{ϕ}}_{1})=\mathrm{Pr}[E_{h}=e_{h}(t)]$$

*Observation 2* $\left({\text{ϕ}}_{2}\right)$*:* The operating channel is sensed as free. The SU transmits data but does not receive the ACK, which implies that the PU was misdetected and the transmission resulted in collision with the PU. No throughput is achieved in this case, *i.e.*, $R(e(t),p_{O}(t),p_{B}(t),NS,{\text{ϕ}}_{2})=0$. The updated belief of the operating and backup channels are given, respectively, as

(16a) $$p_{O}(t+1)=P_{O}^{BF}$$

(16b) $$p_{B}(t+1)=\left\{ \begin{array}{l} p_{B}(t)P_{B}^{FF}\text{, }if\;{\hat{H}}_{0,B}^{N}\;\\ (1-p_{B}(t))P_{B}^{BF}\text{, }if\;{\hat{H}}_{1,B}^{N} \end{array}\right.$$

Energy of the SU is updated to $e\left(t+1\right)=min\left(e\left(t\right)+e_{h}\left(t\right)-e_{s}-e_{tx},\;e_{max}\right)$. The observation and transition probabilities are given, respectively, as

(16c) $$\mathrm{Pr}({\text{ϕ}}_{2})=(1-p_{O}(t))(1-Q_{d,j})$$

(16d) $$\mathrm{Pr}(e(t)\to e(t+1)|{\text{ϕ}}_{2})=\mathrm{Pr}[E_{h}=e_{h}(t)]$$

*Observation 3* $\left({\text{ϕ}}_{3}\right)$*:* Both operating channel and backup channel are sensed as busy. The SU prefers not to switch and sleeps for rest of the current time slot. Because there is no transmission in the current time slot, no throughput is achieved, *i.e.*, $R(e(t),p_{O}(t),p_{B}(t),NS,{\text{ϕ}}_{3})=0$. The updated beliefs about the operating and backup channels, respectively, are given as

(17a) $$\begin{array}{l} p_{O}(t+1)=p_{t}P_{O}^{FF}+(1-p_{t})P_{O}^{BF}\;\\ \text{where }p_{t}=\frac{p_{O}(t)Q_{f,j}}{p_{O}(t)Q_{f,j}+(1-p_{O}(t))Q_{d,j}} \end{array}$$

(17b) $$\begin{array}{l} p_{B}(t+1)=p_{t}P_{B}^{FF}+(1-p_{t})P_{B}^{BF}\;\\ \text{where }p_{t}=\frac{p_{B}(t)P_{f}^{B}}{p_{B}(t)P_{f}^{B}+(1-p_{B}(t))P_{d}^{B}} \end{array}$$

Energy of the SU is updated to $e\left(t+1\right)=min\left(e\left(t\right)+e_{h}\left(t\right)-e_{s}-e_{sw},\;e_{max}\right)$. The observation and transition probabilities, respectively, are given as

(17c) $$\mathrm{Pr}({\text{ϕ}}_{3})=(p_{O}(t)Q_{f,j}+(1-p_{O}(t)Q_{d,j})((1-p_{B}(t)(1-P_{d}^{B}))$$

(17d) $$\mathrm{Pr}(e(t)\to e(t+1)|{\text{ϕ}}_{3})=\mathrm{Pr}[E_{h}=e_{h}(t)]$$

#### 4.3.3. Switch (SW) Mode (a(t)=SW):

Two observations are defined for the switch mode based on the sensing results of the channels. When the SU decides to operate in this mode, it gets a different value of throughput depending on the observations as shown below. 

*Observation 1* $\left({\text{ϕ}}_{4}\right)$*:* The operating channel is sensed as busy, whereas the backup channel is sensed as free. The SU switches to the backup channel and transmits data on it. If the transmission is followed by receiving an ACK before timeout from the corresponding receiver, successful transmission is confirmed. The achieved throughput in this case is expressed as

(18a) $$R(e(t),p_{O}(t),p_{B}(t),SW,{\text{ϕ}}_{4})=\frac{T-t_{s}-t_{sw}}{T}C$$

The updated beliefs about the operating and backup channels, respectively, are given as

(18b) $$\begin{array}{l} p_{O}(t+1)=p_{t}P_{O}^{FF}+(1-p_{t})P_{O}^{BF}\;\\ \text{where }p_{t}=\frac{p_{O}(t)Q_{f,j}}{p_{O}(t)Q_{f,j}+(1-p_{O}(t))Q_{d,j}} \end{array}$$

(18c) $$p_{B}(t+1)=P_{B}^{FF}$$

Energy of the SU is updated to $e\left(t+1\right)=min\left(e\left(t\right)+e_{h}\left(t\right)-e_{s}-e_{sw}-e_{tx},\;e_{max}\right)$. The observation and transition probabilities, respectively, are given as

(18d) $$\mathrm{Pr}({\text{ϕ}}_{4})=(p_{O}(t)Q_{f,j}+(1-p_{O}(t)Q_{d,j})(p_{B}(t)(1-P_{f}^{B}))$$

(18e) $$\mathrm{Pr}(e(t)\to e(t+1)|{\text{ϕ}}_{4})=\mathrm{Pr}[E_{h}=e_{h}(t)]$$

*Observation 2* $\left({\text{ϕ}}_{5}\right)$*:* The operating channel is sensed as busy, and the backup channel is sensed as free. The SU transmits on the backup channel. However, the ACK is not received, indicating collision between the SU transmission and the PU. Due to unsuccessful transmission, no throughput is achieved in this case, *i.e.*, $R(e(t),p_{O}(t),p_{B}(t),SW,{\text{ϕ}}_{5})=0$. The updated beliefs of the operating and backup channels, respectively, are given as

(19a) $$\begin{array}{l} p_{O}(t+1)=p_{t}P_{O}^{FF}+(1-p_{t})P_{O}^{BF}\;\\ \text{where }p_{t}=\frac{p_{O}(t)Q_{f,j}}{p_{O}(t)Q_{f,j}+(1-p_{O}(t))Q_{d,j}} \end{array}$$

(19b) $$p_{B}(t+1)=P_{B}^{BF}$$

Energy of the SU is updated to $e\left(t+1\right)=min\left(e\left(t\right)+e_{h}\left(t\right)-e_{s}-e_{sw}-e_{tx},\;e_{max}\right)$. The observation and transition probabilities, respectively, are given as

(19c) $$\mathrm{Pr}({\text{ϕ}}_{5})=(p_{O}(t)Q_{f,j}+(1-p_{O}(t)Q_{d,j})((1-p_{B}(t)(1-P_{d}^{B}))$$

(19d) $$\mathrm{Pr}(e(t)\to e(t+1)|{\text{ϕ}}_{5})=\mathrm{Pr}[E_{h}=e_{h}(t)]$$

It is noteworthy that the summation of the probabilities of observation should be less than or equal to unity, *i.e.*, $\overset{5}{\underset{i=1}{\sum }}\text{Pr}({\text{ϕ}}_{i})\leq 1$. Proof is given in Appendix A. A summary of the above observations is given in Table 1. According to the above observations, the value function in Equation (13) can be written as

(20) $$V(e(t),p_{O}(t),p_{B}(t))=\underset{a(t)\in A}{\max}\left\{\begin{array}{l} \sum_{t=k}^{\infty }{\text{δ}}^{t-k}\sum_{i=1}^{5}\mathrm{Pr}({\text{ϕ}}_{i})\\ \sum_{e(t+1)}\left[\begin{array}{l} \mathrm{Pr}(e(t)\to e(t+1)|{\text{ϕ}}_{i})\\ R(e(t),p_{O}(t),p_{B}(t),a(t)|{\text{ϕ}}_{i})\\ \left|e(k),p_{O}(k),p_{B}(k\right) \end{array}\right] \end{array}\right\}$$

**Figure 4:**
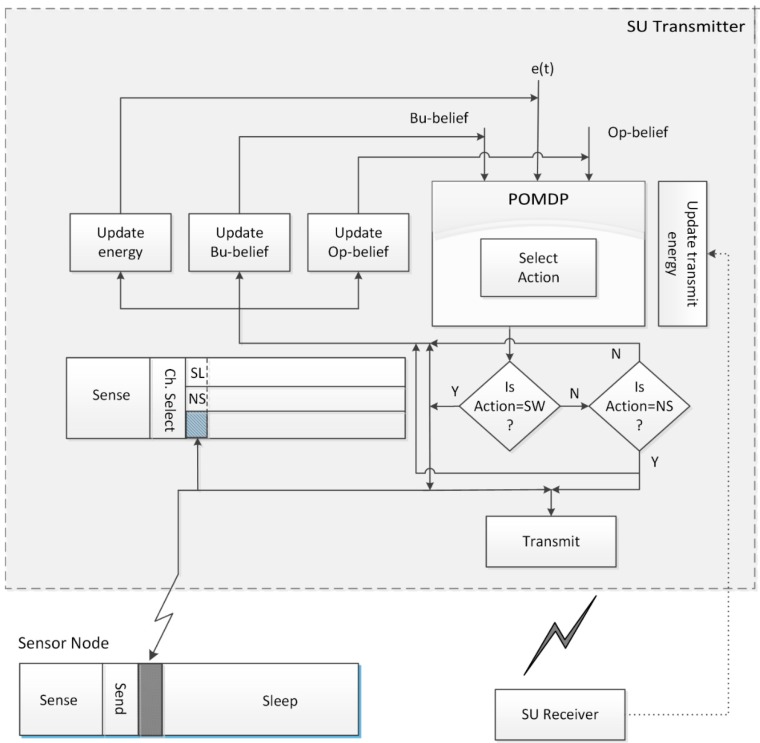
Flowchart of the proposed system.

The optimization problem in the above equation can be solved by the value iteration method [45] to find an optimal decision for maximizing throughput of the SU. Operation of the proposed system is summarized in the flowchart in Figure 4.

**Table 1:** Summary of the throughput, observations, and observation probabilities.

Operation Mode/Action	Throughput	Observation	Probability of Observation ($Pr({\text{ϕ}}_{i})$
**Sleep (SL)**	Zero	Insufficient energy	
**No Switch (NS)**	$\frac{T-\tau_{s}}{T}C$	Op = Free, ACK (Transmit Data ↔ receive ACK), Bu = Don’t care	$\mathrm{Pr}({\text{ϕ}}_{1})=p_{O}(t)(1-Q_{f,j})$
	Zero	Op = Free, NACK (Transmit Data ↔ receive ACK) Bu = Don’t care	$\mathrm{Pr}({\text{ϕ}}_{2})=(1-p_{O}(t))(1-Q_{d,j})$
	Zero	Op = Busy, Bu = Busy	$\begin{array}{c} \mathrm{Pr}({\text{ϕ}}_{3})=(p_{O}(t)Q_{f,j}+(1-p_{O}(t))Q_{d,j})*\\ \;(p_{B}(t)P_{f}^{B}+(1-p_{B}(t))P_{d}^{B}) \end{array}$
**Switch (SW)**	$\frac{T-t_{s}-t_{sw}}{T}C$	Op = Busy Bu = Free, ACK	$\begin{array}{c} \mathrm{Pr}({\text{ϕ}}_{4})=(p_{O}(t)Q_{f,j}+(1-p_{O}(t))Q_{d,j})*\\ \;(p_{B}(t)(1-P_{f}^{B})) \end{array}$
	Zero	Op = Busy Bu = Free, NACK	$\begin{array}{c} \mathrm{Pr}({\text{ϕ}}_{5})=(p_{O}(t)Q_{f,j}+(1-p_{O}(t))Q_{d,j})*\\ \;((1-p_{B}(t)(1-P_{d}^{B})) \end{array}$

## 5. Results and Discussion

The performance of the proposed handoff scheme, which is described in terms of collision probability and throughput against different parameters, has been measured through extensive simulations and compared with “POMDP–no backup [13]”, “Myopic [46]”, and “Myopic-optimal” schemes. The POMDP–no backup scheme makes an optimal decision to stay idle or sense/transmit based on POMDP, but considers only the operating channel for transmission, whereas the Myopic and Myopic-optimal schemes consider only the current time slot for the value function. The Myopic scheme uses an arbitrary sensing time while the Myopic-optimal scheme uses an optimal sensing time. The POMDP-based scheme considers the entire future horizon to maximize the value function. The parameters used for simulations, unless otherwise specified, are summarized in Table 2. The simulation is executed for 2000 iterations (slots).

**Table 2:** Simulation parameters.

Description	Symbol	Value
Number of sensor nodes	*N* − 1	15
Number of channels	*C*	5
Operating (backup) channel idle probability	$P(H_{0,j})=\;P(H_{0,B})$	0.5
Transition probability from state F to itself (for backup and operating channels)	$P_{O}^{FF}=P_{B}^{FF}$	0.7
Transition probability from state B to F (for backup and operating channels)	$P_{O}^{BF}=P_{B}^{BF}$	0.3
Signal-to-noise ratio at SU for *C*-1 channels	$\;{\text{γ}}_{N}$	−10 dB
Signal-to-noise ratio at sensor nodes	${\text{γ}}_{l},\;(l=1,..,N-1)$	−20 dB *to* − 5 dB
Slot duration	T	30 ms
Sensing duration	$t_{s}$	1 ms
Switching duration	$t_{sw}$	500 μs
Battery maximum capacity	$e_{max}$	4
Mean harvested energy	$e_{h\mu }$	2
Transmission energy	$e_{tx}$	2
Energy consumed in sensing	$e_{s}$	1
Energy consumed in switching	$e_{sw}$	0.5
Discount factor	δ	0.99

**Figure 5:**
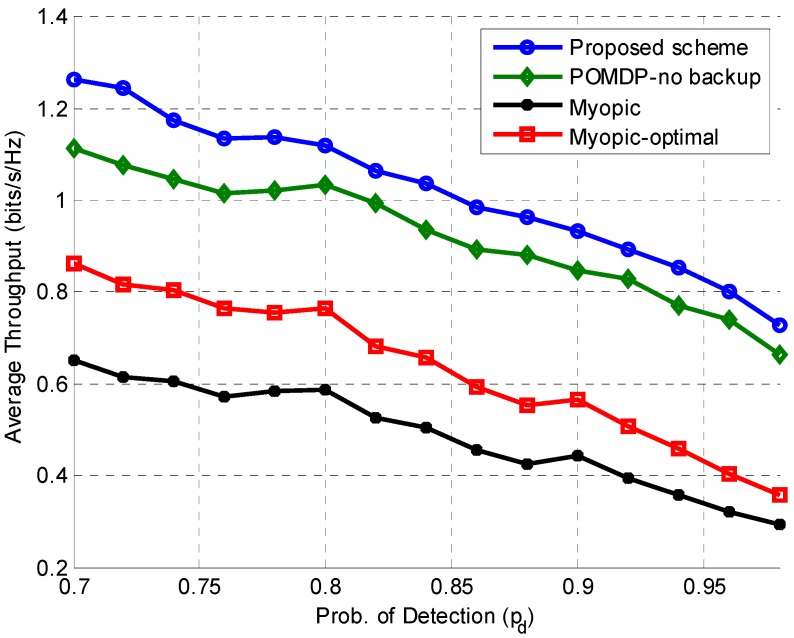
Average throughput comparison of our proposed scheme with POMDP–no backup, Myopic, and Myopic-optimal schemes for different values of $P_{d}$.

Figure 5 shows the average throughput of the proposed scheme in comparison with POMDP–no backup, Myopic-optimal, and Myopic schemes for different values of the target detection probability $\left(P_{d}\right)$. A high value of detection probability indicates more protection for the PU, and thus fewer transmission opportunities for the SU, which translate into reduced throughput of the secondary network. This relationship is clearly visible in the figure, as the throughput decreases for increasing values of target detection probability. For all values of the detection probability, the proposed scheme performs better in terms of achieving higher average throughput compared with the other schemes by taking an energy-efficient POMDP-based optimal decision to switch to the backup channel for transmission or to stay idle on the operating channel.

**Figure 6:**
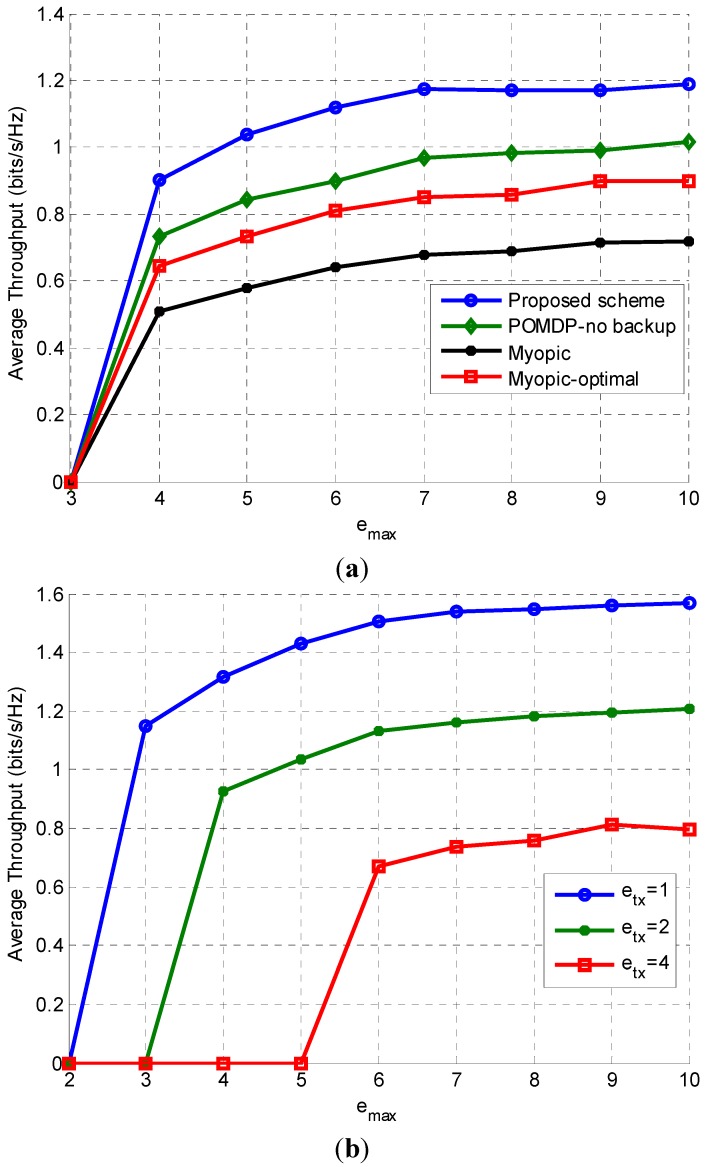
(**a**) Effect of battery capacity on average throughput when $P_{d}=0.9,\;p_{0}=p_{1}=0.5,\;\;e_{t}=2,\;e_{s}=e_{sw}=0.5,\;e_{mean}=2$; (**b**) Effect of transmission energy on average throughput for different values of $e_{max}$.

The results of the investigation into maximum average throughput for different battery capacities are presented in Figure 6a,b. It is logical to expect the throughput to increase with increasing battery capacity, as shown in Figure 6a, because a higher value for $e_{max}$ means there is more energy available to the SU for transmission. The proposed scheme results in the highest average throughput values for the given battery capacities ($e_{max}$) because of its energy-efficient decisions. In Figure 6b, average throughput of the proposed scheme is plotted against $e_{max}$ for different values of transmission energy. It is observed that average throughput decreases with increasing transmission energy, as the SU spends more time in sleep mode due to insufficient energy for sensing, transmission, and/or switching. It is noteworthy that for $e_{tx}=1$, $e_{tx}=2$, and $e_{tx}=4$, there is no throughput for $e_{max}$ less than 2, 3, and 5, respectively. The reason is that the required energy for sensing, transmission, and switching exceeds the maximum capacity.

**Figure 7:**
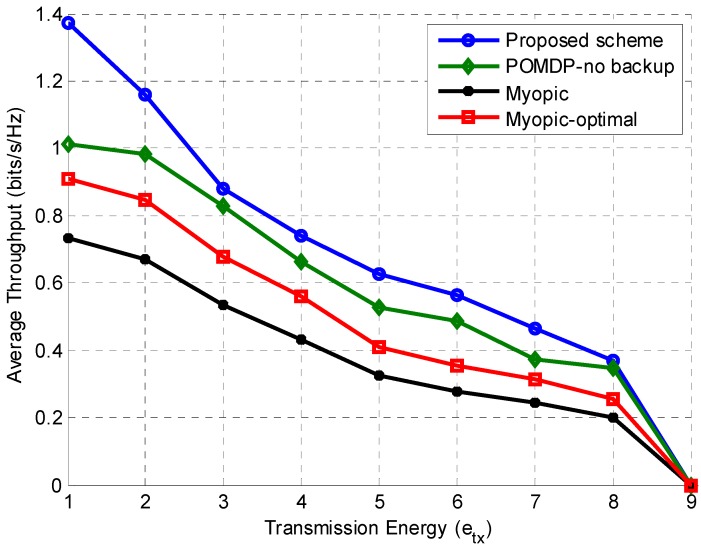
Throughput comparison of different schemes according to the transmission energy ($e_{tx}$) when $e_{max}=10$.

Figure 7 provides further insight into the effects of transmission energy on average throughput. The proposed scheme utilizes the backup channel and gives the best throughput for all values of $e_{tx}$ among all the schemes considered in this study. The value of $e_{max}$ is fixed at 10, while the value of transmission energy is increased. The throughput drops to zero for $e_{tx}=9$ because the transmission and sensing energy become equal to $e_{max}$, which is the condition for no transmission.

**Figure 8:**
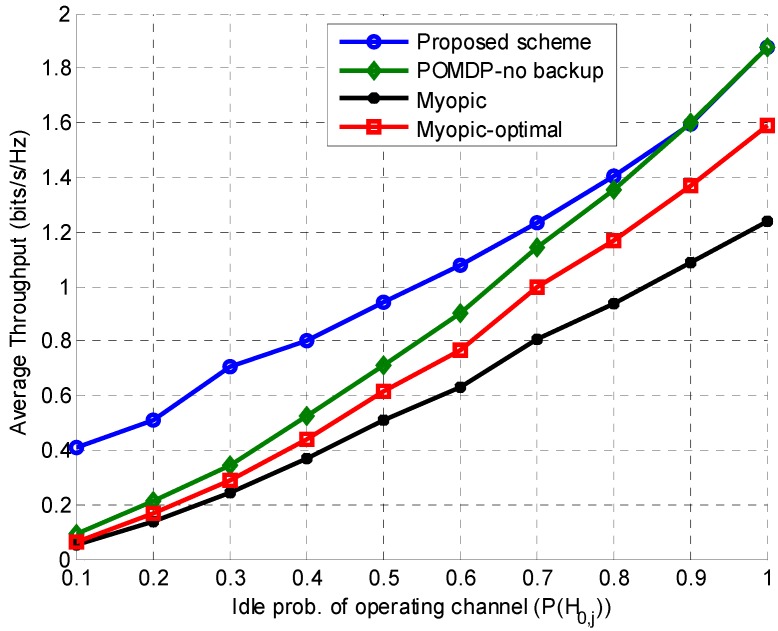
Effect of the operating channel idle probability on average throughput while keeping the backup channel idle probability fixed.

Figure 8 presents the effects of the idle probability (belief) of the operating channel on average throughput. The idle probability of the operating channel is varied, while keeping the idle probability of the backup channel fixed at 0.5. For $P(H_{0,j})<0.5$, the SU transmits on the backup channel because transmission on the operating channel results in significantly lower throughput. When $P(H_{0,j})$ is increased from 0.5 to 0.8, throughput of the POMDP–no backup, Myopic-optimal. and Myopic schemes increases, but remains less than that of the proposed scheme because the proposed scheme continues transmission by switching to the backup channel when the operating channel is busy (with $P(H_{0,B})=0.5$). For $P(H_{0,j})>0.8$, the probability of the operating channel being busy becomes very low, and the SU remains on the operating channel resulting in almost the same throughput as on the operating channel.

**Figure 9:**
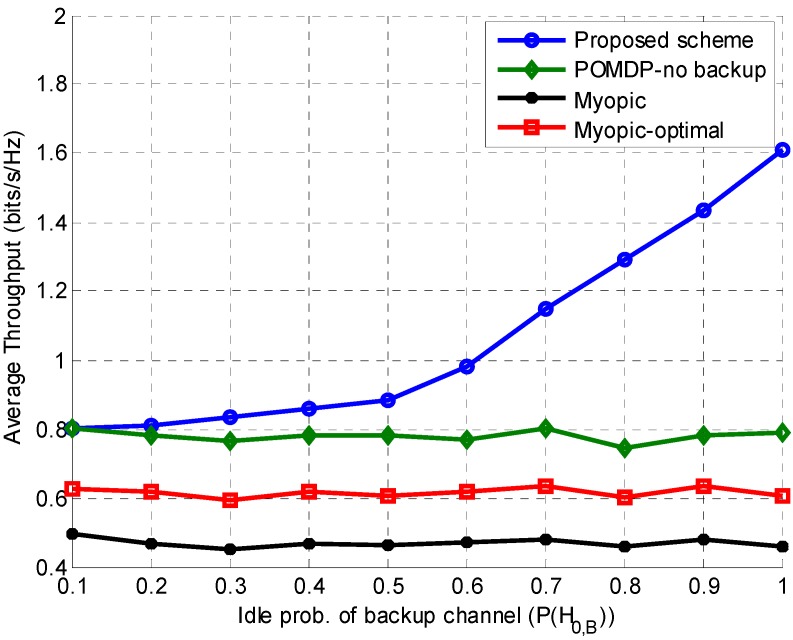
Effect of the backup channel idle probability on average throughput while keeping the operating channel idle probability fixed.

In Figure 9, the average throughput of the SU is studied by varying the idle probability of the backup channel ($P(H_{0,B})$) while keeping the idle probability of the operating channel ($P(H_{0,j})$) fixed at 0.5. Since the value of $P(H_{0,j})$ does not change, throughput of the other schemes remains constant. On the other hand, when $P(H_{0,B})<0.5$, the SU switches to the backup channel for transmission when the operating channel is busy, thus increasing throughput of the proposed scheme. For $P(H_{0,B})>0.5$, the SU mostly remains on the backup channel due to its high availability. Comparison of Figure 8 and Figure 9 reveals a similar general trend in average throughput (*i.e.*, it increases with the increasing idle probabilities of operating or backup channels). However, it can be seen that the average throughput corresponding to the highest value of $P(H_{0,j})$ is higher than that of the highest value of $P(H_{0,B})$. This is because a higher value of $P(H_{0,B})$ compels the SU to switch to the backup channel, costing extra energy and delay, which ultimately causes slightly lower throughput.

**Figure 10:**
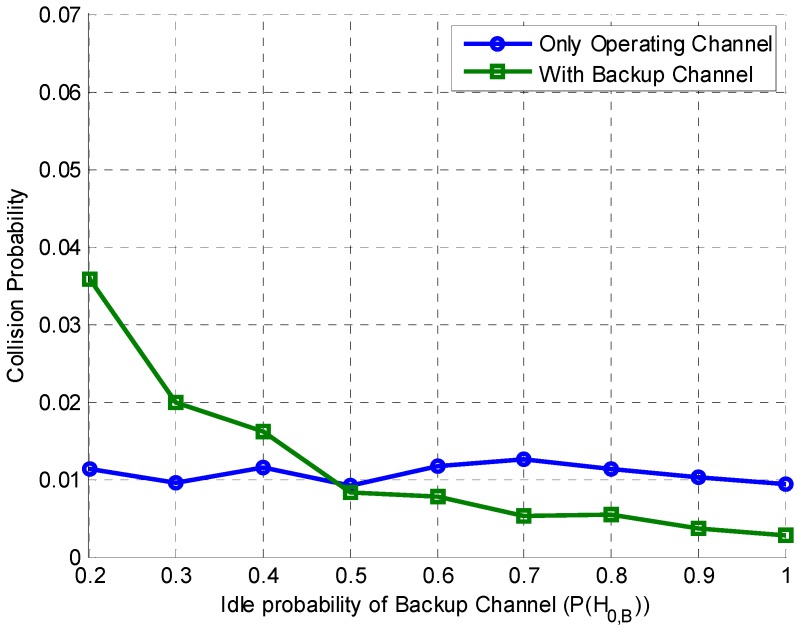
Collision probability of the POMDP-based channel handoff with the backup channel and without the backup channel (operating channel only).

A comparison of the collision probabilities for the proposed scheme and POMDP–no backup scheme is given in Figure 10. Since, in this paper, a slotted operation of the SU is considered, collision with the PU occurs when (i) the SU fails to detect the PU on the operating channel and transmits (observation 2, no switch mode); (ii) the SU detects the PU on the operating channel and switches to the backup channel, where it fails to detect the PU and transmits (observation 2, switch mode); and (iii) the PU appears in the middle of the SU transmission time slot on either the operating or backup channels. Collision in the latter is insignificant due to time-slotted operation of the SU because, in the subsequent time slot, the channel would be recognized as busy, and the SU would refrain from further transmission. Therefore, the collision probability between SU and PU is given as

(21a) $$P_{CO}=(1-P(H_{0,j}))(1-Q_{d,j})$$

(21b) $$P_{CB}=(1-P(H_{0,j}))(1-Q_{d,j})*(1-P(H_{0,B}))(1-P_{d}^{B})$$

where $P_{CO}$ and $P_{CB}$ are the collision probabilities of the operating and backup channels, respectively. It is clear from Figure 10 that the collision probability of the POMDP–no backup scheme remains constant (1%) because of the fixed idle (busy) probability of the operating channel (0.5). The collision probability of the proposed scheme varies with the idle probability of the backup channel. When the idle probability $P(H_{0,B})$ is low, the PU is busy most of the time and the chances of misdetection are high, leading to a slightly higher collision probability. The collision probability decreases with increasing $P(H_{0,B})$ because the chances of misdetection decrease as the busy states of the PU decrease.

## 6. Conclusions and Future Work

Protection of the PU is the primary concern of a secondary network. When a PU appears on a channel, the SU needs to instantly vacate the occupied channel and look for another vacant channel. At the same time, disruption in the SU transmission should be minimized in order to guarantee QoS of the SU. Utilization of the secondary network significantly improves if a backup channel is considered in addition to an operating channel. Transmission on the backup channel enhances throughput of the SU when the operating channel is busy. In this work, an energy-efficient channel handoff scheme is proposed to decide “Should we switch the channel?” The optimal decision is taken using the POMDP framework based upon residual energy and availability of operating and backup channels. A practical scenario with errors in sensing is considered in the analysis. The proposed system maximizes throughput of the SU and causes fewer collisions with the PU.

Future extensions of this work will consider full-duplex cognitive radios, and the integration of cloud computing services with the current system will also be considered. Another potential extension of this work is to address security vulnerabilities in the current scheme (e.g., investigation of false channel handoffs, which occur if an SU behaves maliciously by pretending to be a PU and forces another SU to perform handoff).

## Appendix A

The summation of probabilities of observations should be less than or equal to unity. *i.e.*, $\sum_{i=1}^{5}\mathrm{Pr}({\text{ϕ}}_{i})\leq 1$.

(A1) $$\sum_{i=1}^{5}\mathrm{Pr}({\text{ϕ}}_{i})=\mathrm{Pr}({\text{ϕ}}_{1})+\mathrm{Pr}({\text{ϕ}}_{2})+\mathrm{Pr}({\text{ϕ}}_{3})+\mathrm{Pr}({\text{ϕ}}_{4})+\mathrm{Pr}({\text{ϕ}}_{5})$$

Putting the observations probabilities, defined in Section 4, in Equation (A1).

(A2) $$\begin{array}{l} =p_{O}(t)(1-Q_{f,j})\\ \;+\;(1-p_{O}(t))(1-Q_{d,j})\\ \;+\;(p_{O}(t)Q_{f,j}+(1-p_{O}(t))Q_{d,j})*(p_{B}(t)P_{f}^{B}+(1-p_{B}(t))P_{d}^{B})\\ \;+\;(p_{O}(t)Q_{f,j}+(1-p_{O}(t))Q_{d,j})*(p_{B}(t)(1-P_{f}^{B}))\\ \;+\;(p_{O}(t)Q_{f,j}+(1-p_{O}(t))Q_{d,j})*((1-p_{B}(t)(1-P_{d}^{B})) \end{array}$$

After simplifying and re-arranging Equation (A2), the following is obtained.

(A3) $$\begin{array}{l} =p_{O}(t)-p_{O}(t)-p_{O}(t)Q_{f,j}+p_{O}(t)Q_{f,j}+1-Q_{d,j}+Q_{d,j}\\ \;+\;p_{O}(t)Q_{d,j}-p_{O}(t)Q_{d,j}+p_{O}(t)Q_{f,j}p_{B}(t)P_{f}^{B}-p_{O}(t)Q_{f,j}p_{B}(t)P_{f}^{B}\\ \;+\;p_{O}(t)Q_{f,j}P_{d}^{B}-p_{O}(t)Q_{f,j}P_{d}^{B}-p_{O}(t)Q_{f,j}p_{B}(t)P_{d}^{B}+p_{O}(t)Q_{f,j}p_{B}(t)P_{d}^{B}\\ \;+\;Q_{d,j}p_{B}(t)P_{f}^{B}-Q_{d,j}p_{B}(t)P_{f}^{B}+Q_{d,j}P_{d}^{B}-Q_{d,j}P_{d}^{B}-Q_{d,j}p_{B}(t)P_{d}^{B}+Q_{d,j}p_{B}(t)P_{d}^{B}\\ \;-\;p_{O}(t)Q_{d,j}p_{B}(t)P_{f}^{B}+p_{O}(t)Q_{d,j}p_{B}(t)P_{f}^{B}-p_{O}(t)Q_{d,j}P_{d}^{B}+p_{O}(t)Q_{d,j}P_{d}^{B}\\ \;+\;p_{O}(t)Q_{d,j}p_{B}(t)P_{d}^{B}-p_{O}(t)Q_{d,j}p_{B}(t)P_{d}^{B}+p_{O}(t)Q_{f,j}p_{B}(t)-p_{O}(t)Q_{f,j}p_{B}(t)\\ \;+\;Q_{d,j}p_{B}(t)-Q_{d,j}p_{B}(t)-p_{O}(t)Q_{d,j}p_{B}(t)+p_{O}(t)Q_{d,j}p_{B}(t)\\ =\;1 \end{array}$$

## References

[1] Federal Communications Commission (FCC) (2002). Report of the Spectrum Efficiency Working Group.

[2] Weiss M.B., Delaere S., Lehr W.H. Sensing as a Service: An Exploration into Practical Implementations of DSA. Proceedings of the IEEE International Symposium on New Frontiers in Dynamic Spectrum Access Networks (DySPAN 2010).

[3] Federal Communications Commission (FCC) (2010). In the Matter of: Promoting More Efficient Use of Spectrum Through Dynamic Spectrum Use Technologies (ET Docket No. 10-237). 10-198: Notice of Inquiry.

[4] Mercier B., Fodor V., Thobaben R., Skoglund M., Koivunen V., Lindfors S., Ryynänen J., Larsson E.G., Petrioli C., Bongiovanni G. Sensor networks for cognitive radio: Theory and system design. Proceedings of the ICT Mobile Summit.

[5] Usman M., Har D., Koo I. Energy-Efficient Infrastructure Sensor Network for *Ad Hoc* Cognitive Radio Network. IEEE Trans. Ind. Inform.

[6] Deng R., Chen J., Yuen C., Cheng P., Sun Y. (2012). Energy-efficient cooperative spectrum sensing by optimal scheduling in sensor-aided cognitive radio networks. IEEE Trans. Veh. Technol..

[7] Mishra S.M., Sahai A., Brodersen R.W. Cooperative sensing among cognitive radios. Proceedings of the IEEE International Conference on Communications (ICC ’06).

[8] Ghasemi A., Sousa E.S. Collaborative spectrum sensing for opportunistic access in fading environments. New Frontiers in Dynamic Spectrum Access Networks; Proceedings of the First IEEE International Symposium on (DySPAN 2005).

[9] Wang C.-W., Wang L.-C. (2012). Analysis of reactive spectrum handoff in cognitive radio networks. IEEE J. Sel. Areas Commun..

[10] Ma R.-T., Hsu Y.-P., Feng K.-T. A POMDP-based spectrum handoff protocol for partially observable cognitive radio networks. Proceedings of the IEEE Wireless Communications and Networking Conference (WCNC 2009).

[11] Wang L.-C., Wang C.-W. Spectrum handoff for cognitive radio networks: Reactive-sensing or proactive-sensins?. Proceedings of the IEEE International Performance, Computing and Communications Conference (IPCCC).

[12] Hoang A.T., Liang Y.-C., Wong D.T.C., Zeng Y., Zhang R. (2009). Opportunistic spectrum access for energy-constrained cognitive radios. IEEE Trans. Wireless Commun..

[13] Sultan A. (2012). Sensing and transmit energy optimization for an energy harvesting cognitive radio. Wireless Commun. Lett. IEEE.

[14] Vu-Van H., Koo I. (2014). Optimal Throughput for Cognitive Radio with Energy Harvesting in Fading Wireless Channel. Sci. World J..

[15] Lee D., Yeo W. (2015). Channel Availability Analysis of Spectrum Handoff in Cognitive Radio Networks. IEEE Commun. Lett..

[16] Sheikholeslami F., Nasiri-Kenari M., Ashtiani F. (2015). Optimal Probabilistic Initial and Target Channel Selection for Spectrum Handoff in Cognitive Radio Networks. IEEE Trans. Wireless Commun..

[17] Shokri-Ghadikolaei H., Glaropoulos I., Fodor V., Fischione C., Dimou K. (2013). Energy Efficient Spectrum Sensing and Handoff Strategies in Cognitive Radio Networks.

[18] Zhang Y. Spectrum handoff in cognitive radio networks: Opportunistic and negotiated situations. Proceedings of the IEEE International Conference on Communications (ICC ’09).

[19] Wang S., Wang Y., Coon J.P., Doufexi A. (2012). Energy-efficient spectrum sensing and access for cognitive radio networks. IEEE Trans. Veh. Technol..

[20] Han J.A., Jeon W.S., Jeong D.G. (2011). Energy-efficient channel management scheme for cognitive radio sensor networks. IEEE Trans. Veh. Technol..

[21] Lertsinsrubtavee A., Malouch N., Fdida S. Controlling spectrum handoff with a delay requirement in cognitive radio networks. Proceedings of the 21st International Conference on Computer Communications and Networks (ICCCN).

[22] Zeeshan M., Manzoor M.F., Qadir J. Backup channel and cooperative channel switching on-demand routing protocol for multi-hop cognitive radio ad hoc networks (BCCCS). Proceedings of the 6th International Conference on Emerging Technologies (ICET).

[23] Wang J.-W., Adriman R. Analysis of cognitive radio networks with imperfect sensing and backup channels. Proceedings of the 7th International Conference on Innovative Mobile and Internet Services in Ubiquitous Computing (IMIS).

[24] Kalil M.A., Al-Mahdi H., Mitschele-Thiel A. Spectrum handoff reduction for cognitive radio ad hoc networks. Proceedings of the 7th International Symposium on Wireless Communication Systems (ISWCS).

[25] Chengyu W., Chen H., Lingge J. (2013). Spectrum handoff scheme based on recommended channel sensing sequence. Commun. China.

[26] Mishra V., Lau C.T., Chan S. (2013). Reconfigurable channel selection technique for cognitive radio network with heterogeneous primary bands. IEEE Trans. Veh. Technol..

[27] Song Y., Xie J. (2012). ProSpect: A proactive spectrum handoff framework for cognitive radio ad hoc networks without common control channel. IEEE Trans. Mob. Comput..

[28] Liu Y., Yuen C., Ul Hassan N., Huang S., Yu R., Xie S. (2015). Electricity cost minimization for a microgrid with distributed energy resource under different information availability. IEEE Trans. Ind. Electron..

[29] Sun H., Nallanathan A., Wang C.-X., Chen Y. (2013). Wideband spectrum sensing for cognitive radio networks: A survey. IEEE Wireless Commun..

[30] Quan Z., Cui S., Sayed A.H., Poor H.V. Wideband spectrum sensing in cognitive radio networks. Proceedings of the IEEE International Conference on Communications (ICC ’08).

[31] Li X., Wang D., Mao X., McNair J. (2013). On the Accuracy of Maximum Likelihood Estimation for Primary User Behavior in Cognitive Radio Networks. IEEE Commun. Lett..

[32] Ma J., Li Y. Soft Combination and Detection for Cooperative Spectrum Sensing in Cognitive Radio Networks. Proceedings of the 50th IEEE Global Telecommunications Conference (GLOBECOM ’07).

[33] Liu V., Parks A., Talla V., Gollakota S., Wetherall D., Smith J.R. (2013). Ambient backscatter: Wireless communication out of thin air. ACM SIGCOMM Comput. Commun. Rev..

[34] Lee S., Zhang R., Huang K. (2013). Opportunistic wireless energy harvesting in cognitive radio networks. IEEE Trans. Wireless Commun..

[35] Chen X., Yuen C., Zhang Z. (2014). Wireless energy and information transfer tradeoff for limited-feedback multiantenna systems with energy beamforming. IEEE Trans. Veh. Technol..

[36] Usman M., Koo I. (2014). Access Strategy for Hybrid Underlay-Overlay Cognitive Radios with Energy Harvesting. IEEE Sens. J..

[37] Li Q., Naing V., Donelan J.M. (2009). Development of a biomechanical energy harvester. J. Neuroeng. Rehabil..

[38] Schuss C., Rahkonen T. Solar energy harvesting strategies for portable devices such as mobile phones. Proceedings of the 14th Conference of Open Innovations Association (FRUCT).

[39] Kalyanaraman K., Babu J. Power Harvesting System in Mobile Phones and Laptops using Piezoelectric Charge Generation. Proceedings of the World Congress on Engineering and Computer Science (WCECS).

[40] Wan Z., Tan Y., Yuen C. Review on energy harvesting and energy management for sustainable wireless sensor networks. Proceedings of the 13th IEEE International Conference on Communication Technology (ICCT).

[41] Mateu L., Moll F. (2005). Review of energy harvesting techniques and applications for microelectronics (Keynote Address). Microtechnologies for the New Millennium.

[42] Paradiso J., Starner T. (2005). Energy scavenging for mobile and wireless electronics. IEEE Pervasive Comput..

[43] Chalasani S., Conrad J.M. A survey of energy harvesting sources for embedded systems. Proceedings of the IEEE Southeastcon.

[44] Wang X., Gong J., Hu C., Zhou S., Niu Z. (2015). Optimal power allocation on discrete energy harvesting model. EURASIP J. Wireless Commun. Netw..

[45] Bertsekas D.P. (2001). Dynamic Programming and Optimal Control.

[46] Park S., Heo J., Kim B., Chung W., Wang H., Hong D. Optimal mode selection for cognitive radio sensor networks with RF energy harvesting. Proceedings of the IEEE 23rd International Symposium on Personal Indoor and Mobile Radio Communications (PIMRC).

